# Fear conditioning and fear generalization in children and adolescents with anxiety disorders

**DOI:** 10.1007/s00787-023-02304-7

**Published:** 2023-10-04

**Authors:** Julia Reinhard, Anna Mittermeier, Lisa Brandstetter, Kimberly Mowat, Anna Slyschak, Andrea M. F. Reiter, Matthias Gamer, Marcel Romanos

**Affiliations:** 1 Center of Mental Health, Department of Child and Adolescent Psychiatry, Psychosomatics and Psychotherapy, University Hospital of Würzburg, Würzburg, Germany; 2 Department of Psychology (Experimental Clinical Psychology), University of Würzburg, Würzburg, Germany

**Keywords:** Fear conditioning, Fear generalization, Overgeneralization, Anxiety disorders, Childhood and adolescence, Development

## Abstract

Overgeneralization of conditioned fear is associated with anxiety disorders (AD). Most results stem from studies done in adult patients, but studies with children are rare, although the median onset of anxiety disorders lies already in childhood. Thus, the goal of the present study was to examine fear learning and generalization in youth participants, aged 10–17 years, with AD (*n* = 39) compared to healthy controls (HC) (*n* = 40). A discriminative fear conditioning and generalization paradigm was used. Ratings of arousal, valence, and US expectancy (the probability of an aversive noise following each stimulus) were measured, hypothesizing that children with AD compared to HC would show heightened ratings of arousal and US expectancy, and decreased positive valence ratings, respectively, as well as overgeneralization of fear. The results indicated that children with AD rated all stimuli as more arousing and less pleasant, and demonstrated higher US expectancy ratings to all stimuli when compared to HC. Thus, rather than displaying qualitatively different generalization patterns (e.g., a linear vs. quadratic slope of the gradient), differences between groups were more quantitative (similar, but parallel shifted gradient). Therefore, overgeneralization of conditioned fear does not seem to be a general marker of anxiety disorders in children and adolescents.

**Supplementary Information:**

The online version contains supplementary material available at 10.1007/s00787-023-02304-7.

## Introduction

Fear in the presence of a real threat is a highly adaptive emotional state, which activates the defensive fear system of the organism [1]. It can, however, become maladaptive and pathological if the fear is unreasonable, excessive, and significantly interferes with daily life routines [2]. Anxiety disorders are highly prevalent [3, 4] and are typically characterized by an early onset [5]. Estimates of the incidence of anxiety disorders in children and adolescents range from 5.3% to 17% [6]. Childhood and adolescent anxiety pose a risk for anxiety disorders in adulthood [7] and pathological anxiety has high comorbidity rates with other disorders, e.g., depression [8]. Moreover, anxiety disorders have broad impacts on both the health care system and particularly affected patients, with long-term negative consequences for child maturation [9], e.g., academic, and vocational underachievement [10] as well as impaired social competency [11]. Thus, advancing our understanding of the pathogenesis of anxiety disorders is essential.

In the development of fear and the pathogenesis of anxiety disorders, fear conditioning and generalization are thought to be central learning mechanisms [12], and thus, have been proposed as translational models of the acquisition of clinically relevant fear [13]. Differential fear conditioning refers to learning that a conditioned stimulus (CS +) predicts an aversive event (the unconditioned stimulus (US)) while another stimulus (CS−) is never followed by the US and predicts safety. During fear generalization, conditioned fear responses extend to stimuli (generalization stimuli (GSs)), which share some perceptual similarity with a conditioned stimulus (CS +), but have never been associated with an aversive stimulus. The generalization gradient, namely the curve or slope, which results from the different fear reactions to one stimulus (CS + , GS1-4, CS−) in relation to the next stimuli, usually changes as a function of reduced similarity between GSs and CS + [14]: A steep, quadratic versus a shallow, linear gradient indicates limited versus strong generalization, respectively.

Abnormalities in fear generalization are discussed as risk factors for anxiety disorders since, for instance, panic disorder [15], post-traumatic stress disorder [16], and generalized anxiety disorder [17] appear to be characterized by enhanced fear generalization in adults with anxiety disorder when compared to healthy controls. These studies further showed that generalization gradients of healthy participants follow quadratic trends, whereas those of patients with anxiety disorders follow linear trends. Other studies, however, failed to detect overgeneralization in anxiety patients [18, 19]. Thus, results are mixed and the role of overgeneralization in the pathogenesis of anxiety disorders has not yet been fully clarified so far [see also [20]. Furthermore, most studies concerning aversive conditioning and fear generalization were conducted with adult patients, whereas less is known about these processes in childhood anxiety. While a study in healthy adults showed that increased fear generalization is predictive of subclinical anxiety levels six months later [21] suggesting that heightened fear generalization is not merely a state marker of pathological anxiety, but also represents a risk factor for the development of anxiety at follow-up, studies conducted with children are important to probe the relevance of these observations found in adults.

The few studies comparing fear conditioning in healthy versus anxious children showed that children and adolescents with pathological anxiety were less able to discriminate between stimuli (CS + vs. CS−) [e.g., 22–24] indicating impaired safety signal learning in anxious children. Other studies demonstrated that adolescents with anxiety disorders generally reveal stronger fear reactions towards the threat stimulus (CS +) compared to healthy adolescents [25]. Interestingly, our recent study comparing fear generalization between healthy adults and children aged 8 to 10 years demonstrated that children showed heightened fear generalization [26], similar to what was found in adult anxiety patients [15, 17]. Furthermore, a study on age differences in fear learning and generalization in healthy 8- to 13-year-old children using one intermediate generalization stimulus (GS) and measures of startle responses and self-report ratings showed that only older children (11–13 years old) demonstrated a decline in response strength from the CS + over the GS to the CS− reminiscent of fear generalization patterns in adults [27]. In contrast, younger children exhibited a u-shaped generalization pattern with larger responses to both the CS + and CS− relative to the GS. Thus, generalization of conditioned fear seems to depend not only on anxiety levels but also on children’s age. Therefore, the question arose if overgeneralization per se is associated with anxiety disorders in children, similar to what was found in adults.

The present study investigated fear conditioning and generalization in children and adolescents with clinically relevant anxiety compared to healthy controls (HC). We used an adaption of the “screaming lady” paradigm by Lau et al., 2008 [25] and measured ratings of valence, arousal, and US expectancy. In the current task, two female faces with neutral facial expressions paired with (CS +) or unpaired without (CS−) a 95-dB loud female scream (US) were used as stimuli. Based on findings in adults as well as on aforementioned findings in children and adolescents, we hypothesized that clinically anxious patients compared to HC would show higher ratings of arousal and US expectancy and lower valence ratings, respectively, during fear learning and generalization. Furthermore, patients with anxiety disorders were expected to demonstrate an overgeneralization of conditioned fear as indicated by more linear trends in contrast to more quadratic trends in HC.

## Methods

### Sample

A total of n = 39 children and adolescents with anxiety disorders (27 female; aged 10 to 17 years; mean age: 14.13 years, SD = 1.98 years) and 40 healthy controls (29 female; aged 10 to 17 years; mean age: 14.18 years, SD = 1.97 years) participated in the study. Groups did not differ relevantly in age (*p* = 0.916) nor sex (*p* = 0.471). Within the group of children and adolescents with anxiety disorders, the following diagnoses were included: Social anxiety disorder (SAD) (*n* = 18), generalized anxiety disorder (GAD) (n = 2), separation anxiety disorder (*n* = 6), panic disorder (PD) (*n* = 1), agoraphobia (*n* = 1), specific anxiety disorder (including school anxiety) (*n* = 2), anxiety and depression combined (*n* = 9).

Healthy controls (HC) were recruited from primary/secondary schools in the greater area of Wuerzburg, Germany. Anxiety patients were inpatients in the Department of Child and Adolescent Psychiatry at the University Hospital of Wuerzburg, Germany, and ICD-10 diagnoses were determined by experienced psychologists. Exclusion criteria were a current or past diagnosis of psychosis and/or current suicidal ideation. Additional exclusion criteria were neurological disorders and/or medical conditions that interfered with the objectives of the study as well as significantly limited visual acuity and/or hearing and severe medical conditions (e.g., severe asthma, diabetes). Additional exclusion criteria for HC were a lifetime DSM-IV axis l disorder (ascertained using the German versions of the Diagnostic Interview for Mental Disorders for Children and Adolescents, Kinder-DIPS [28]). All participants were native German speakers with an IQ ≥ 85 as determined by the German version of the Culture Fair Intelligence Test 2 [29].

The study was approved by the ethical committee of the Medical Faculty of the Julius-Maximilian-University of Würzburg (study number 211/16) and complied with the latest version of the Declaration of Helsinki. All participants as well as their parents gave written informed consent and each family was paid € 30 compensation for their participation.

### Psychometric assessment

All participants completed the German versions of the State-Trait Anxiety Inventory for Children—Trait version (STAIC-T [30]), and of the Anxiety Sensitivity Index for Children (CASI [31]). The STAIC-T is a self-report scale to determine the level of trait anxiety on 20 statements on a three-point Likert scale (1) “almost never”, (2) “sometimes” and (3) “often”, resulting in a sum score between 20 and 60. The CASI is a self-report scale to measure the level of anxiety and fearfulness as a reaction to bodily symptoms on 18 items, describing potential reactions to physical symptoms and anxiety on a three-point Likert scale (1) “never”, (2) “sometimes” and (3) “often”, resulting in a sum score between 18 and 54. As expected, there were significant differences between the groups in STAIC-T (*t* (76) = 4.66, *p* < 0.001; patients: *M* = 42.39, *SD* = 9.14; HC: *M* = 33.83, *SD* = 7.03) as well as CASI scores (*t* (75) = 4.20, *p* < 0.001; patients: *M* = 32.51, *SD* = 6.71; HC: *M* = 26.85, *SD* = 5.05).

### Stimulus material

The stimulus presentation was controlled by using the Presentation software version 17.2 (Neurobehavioral Systems, Inc., Albany, CA, USA).

#### Stimuli (CS + , CS−, GS1-4)

We used a modified version of the “screaming lady paradigm” by Lau et al. [25] (Fig. A.1). In this task, pictures of two actresses with neutral facial expressions were presented (NimStim Face Stimulus Set [32]) that served as either the CS + or the CS−, with one of the two faces being randomly selected as the CS + for each participant. Four generalization stimuli depicting gradual morphs from the CS + to the CS− in 20%-steps (GS1-4) were created using the graphics software Sqirlz Morph Version 2.1 (Xiberpix, Solihull, UK).

#### Unconditioned Stimulus (US)

The US was a 95-dB female scream (International Affective Digital Sounds system) presented simultaneously with a fearful facial expression of the same actress assigned as the CS + .

### Task

The experiment was divided into three consecutive phases: pre-acquisition, acquisition, and generalization separated by ratings. The pre-acquisition phase consisted of four CS + and four CS− presentations while no US appeared. During acquisition, 12 CS + and 12 CS− were presented. The CS + was paired with the US on 10 trials (reinforcement rate: 83%). The generalization phase consisted of 12 CS + , 12 CS−, and 12 of each of the four GSs. In the generalization phase, the reinforcement rate was 50%, thus half of the CS + trials (t = 6) were followed by the US to prevent premature extinction. The CS− and all GSs were never paired with the US.

The CSs and the GSs were presented for 6 s each. The US was presented immediately following the CS + offset for 1.5 s. The inter-trial intervals (ITI) varied from 9–12 s, during which a white fixation cross was displayed centrally on the screen. The stimulus order was pseudo-randomized so that the same stimulus could not appear more than twice in a row. The acquisition and generalization trials were separated into two blocks (Acquisition 1, Acquisition 2, and Generalization 1, Generalization 2, respectively), each containing half of the trials per block, that is 6 presentations per stimulus category. The participants were instructed to passively view the pictures of the female faces, and that an unpleasant sound would be heard occasionally, but they were not informed of the CS−US contingencies.

Following pre-acquisition, and each acquisition (2x) and generalization (2x) block, the participants rated each stimulus on arousal, valence, and US expectancy on the computer screen. The arousal and valence ratings were indicated on 9-point Likert scales, ranging from “very calm” (1) to “very arousing” (9), and “very unpleasant” (1) to “very pleasant” (9), respectively. The US expectancy was recorded in percent on a scale from 1 to 100 in 10% increments, as the probability of an aversive noise following each stimulus (from “certainly not “ (1) to “very certain” (11)).

### Statistical analyses

All statistical analyses were performed with IBM SPSS (Version 25, SPSS Inc.). To verify conditioning effects, we first calculated three separate 2 × 2 × 3 repeated-measures ANOVAs on the ratings (arousal, valence, and US expectancy), with the between-subject factor group (healthy controls (HC) vs. patients with anxiety disorders) and the within-subject factors stimulus type (CS + , CS−) and phase (Pre-acquisition, Acquisition 1, Acquisition 2). To analyze generalization effects, we calculated three separate 2 × 6 × 2 repeated-measures ANOVAs for ratings, again with the between-subject factor group (HC vs. anxiety patients) and the within-subject factors stimulus type (CS + , GS1-4, CS−) and phase (Generalization 1, Generalization 2). To investigate the generalization gradient in more detail, we conducted trend analyses for all three variables (ratings of arousal, valence, US expectancy). Greenhouse–Geisser corrections for non-sphericity were performed where indicated, though uncorrected degrees of freedom are reported for the sake of better readability. In case of significant interaction effects, post-hoc *t*-tests were calculated with *p*-values adjusted according to Bonferroni. Corrected *p*-values and partial *η*^2^ are reported. Alpha was set at 0.05.

## Results

### Pre-Acquisition/Acquisition phases

#### Conditioning effects for the ratings

Significant main effects of stimulus type and phase as well as significant stimulus type x phase interaction effects (Table A.1) on the arousal and US expectancy ratings were observed, indicating that the conditioning procedure was successful. Similarly, for the valence ratings, a significant stimulus type effect and a significant stimulus type x phase interaction effect were found (Table A.1; for descriptive statistics please see Table A.2).

Post hoc tests for the arousal ratings indicated that there were significant differences between the stimuli after Acquisition 1 (*t* (78) = 4.46, *p* < 0.001), and after Acquisition 2 (*t* (78) = 4.84, *p* < 0.001), but not after Pre-Acquisition (*t* (78) = 0.58, *p* = 0.567). Differences were due to higher arousal ratings in response to the CS + , not reductions in the CS− ratings (all *p* ≥ 0.354). Thus, there were significant differences between the CS + ratings after the Pre-Acquisition and Acquisition 1 (*t* (78) = 5.44, *p* < 0.001), and after the Pre-Acquisition and Acquisition 2 (*t* (78) = 5.43, *p* < 0.001) with higher CS + ratings after each Acquisition phase compared to the Pre-Acquisition (Fig. A.2). No significant differences were found between the two Acquisition phases (*p* = 0.264), indicating that the participants had acquired a fear response already in Acquisition 1.

Similarly, for the valence ratings, there were significant differences between the stimuli after Acquisition 1 (*t* (78) = 3.40, *p* = 0.001), and Acquisition 2 (*t* (78) = 4.60, *p* < 0.001), but not after the Pre-Acquisition (*t* (78) = 0.82, *p* = 0.414, Fig. A.2). Again, the differences were significant for the CS + ratings, but not for the CS− ratings (all *p* ≥ 0.247): there were significant differences between the CS + ratings after the Pre-Acquisition and Acquisition 1 (*t* (78) = 3.01, *p* = 0.004) as well as after the Pre-Acquisition and Acquisition 2 (*t* (78) = 3.82, *p* < 0.001), but also between the two acquisition phases (*t* (78) = 2.07, *p* = 0.041).

Likewise, the US expectancy ratings were significantly different between CS + and CS− during Acquisition 1 (*t* (78) = 5.06, *p* < 0.001) and Acquisition 2 (*t* (78) = 8.23, *p* < 0.001), but not during Pre-Acquisition (*t* (78) = 0.89, *p* = 0.377, Fig. A.2), again indicating that conditioning was successful. There were significant differences between the CS + ratings after Pre-Acquisition and Acquisition 1 (*t* (78) = 6.80, *p* < 0.001) and after Pre-Acquisition and Acquisition 2 (*t* (78) = 8.53, *p* < 0.001), as well as between the two acquisition phases for the CS + ratings (*t* (78) = 3.18, *p* = 0.002) and the CS− ratings (*t* (78) = 2.89, *p* = 0.005).

#### Effects of group on fear acquisition

**Fig. 1:**
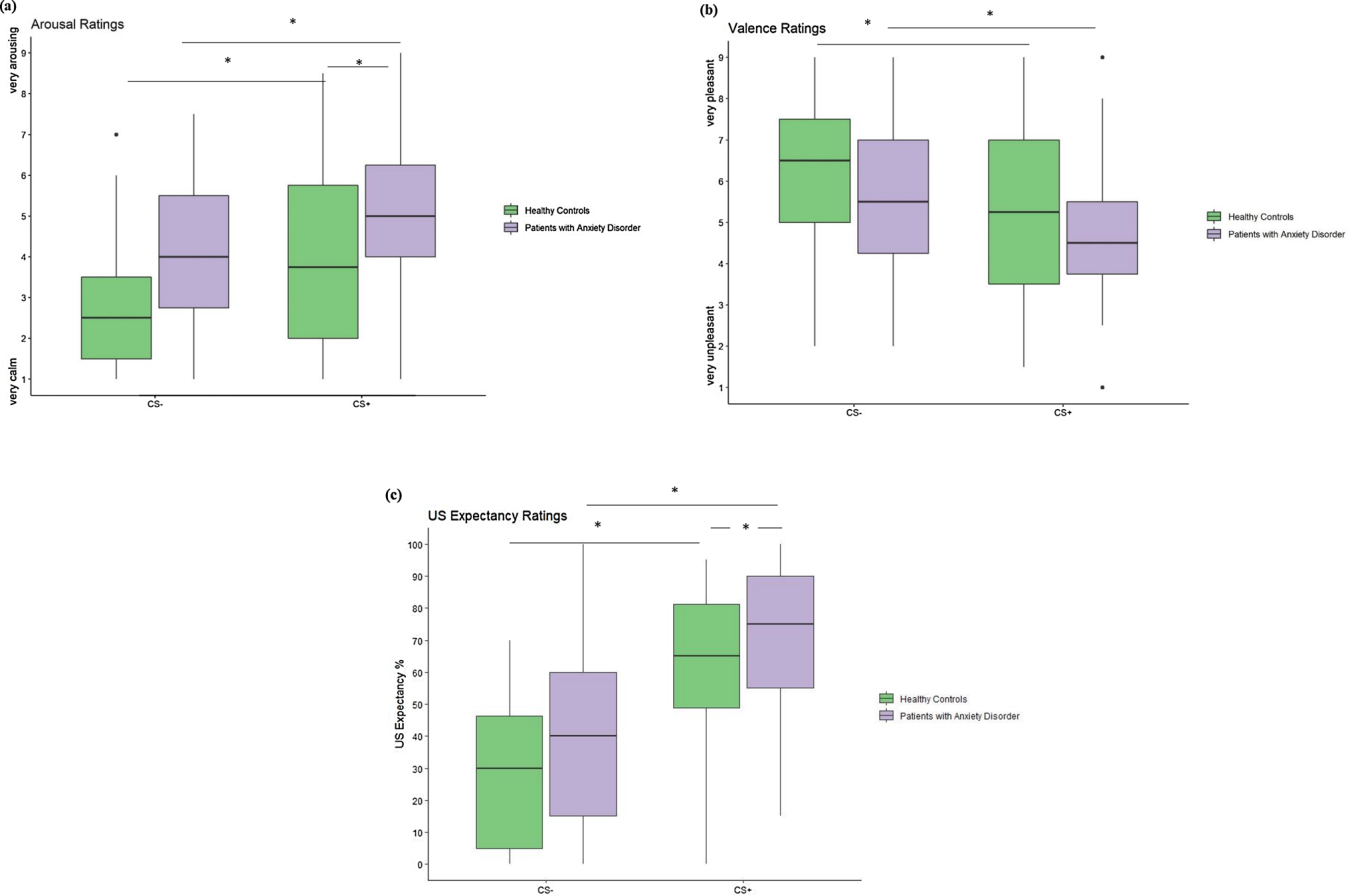
Results during acquisition (the average of acquisition 1 and 2) of **a** arousal, **b** valence, and **c** US expectancy to CS + and CS− by group displayed as boxplots. The lower and upper hinges correspond to the first and third quartiles (i.e., the 25th and 75th percentiles). The horizontal line shows the median value. Whiskers (vertical lines) extend from the hinge to the lowest/largest individual data point, no further than 1.5 * inter-quartile ranges from the hinge. Data beyond the end of the whiskers (i.e., outliers) are plotted individually as points

Significant main effects of the group were found for the arousal (*F* (1,77) = 7.17, *p* = 0.009, *η*^2^ = 0.09) and the US expectancy ratings (*F* (1,77) = 4.34, *p* = 0.040, *η*^2^ = 0.05) indicating overall higher ratings in clinically anxious participants (Fig. 1). No other significant effects concerning group were found (all *p* ≥ 0.051).

### Generalization phases

#### Generalization effects for the ratings

For the arousal, valence, and US expectancy ratings, significant main effects of stimulus type were found (Table A.3; for descriptive statistics please see Table A.4), indicating a downtrend (uptrend, respectively, for valence) from CS + to CS−. Analyses revealed significant linear and quadratic trends for all ratings (Table A.3). Moreover, significant main effects of phase were found for the arousal and the US expectancy ratings (arousal: *F* (1, 77) = 4.34, *p* = 0.041, *η*^2^ = 0.05; US expectancy:* F* (1, 77) = 24.95, *p* < 0.001, *η*^2^ = 0.25) with overall higher ratings after Generalization 1.

#### Effects of group on fear generalization

**Fig. 2:**
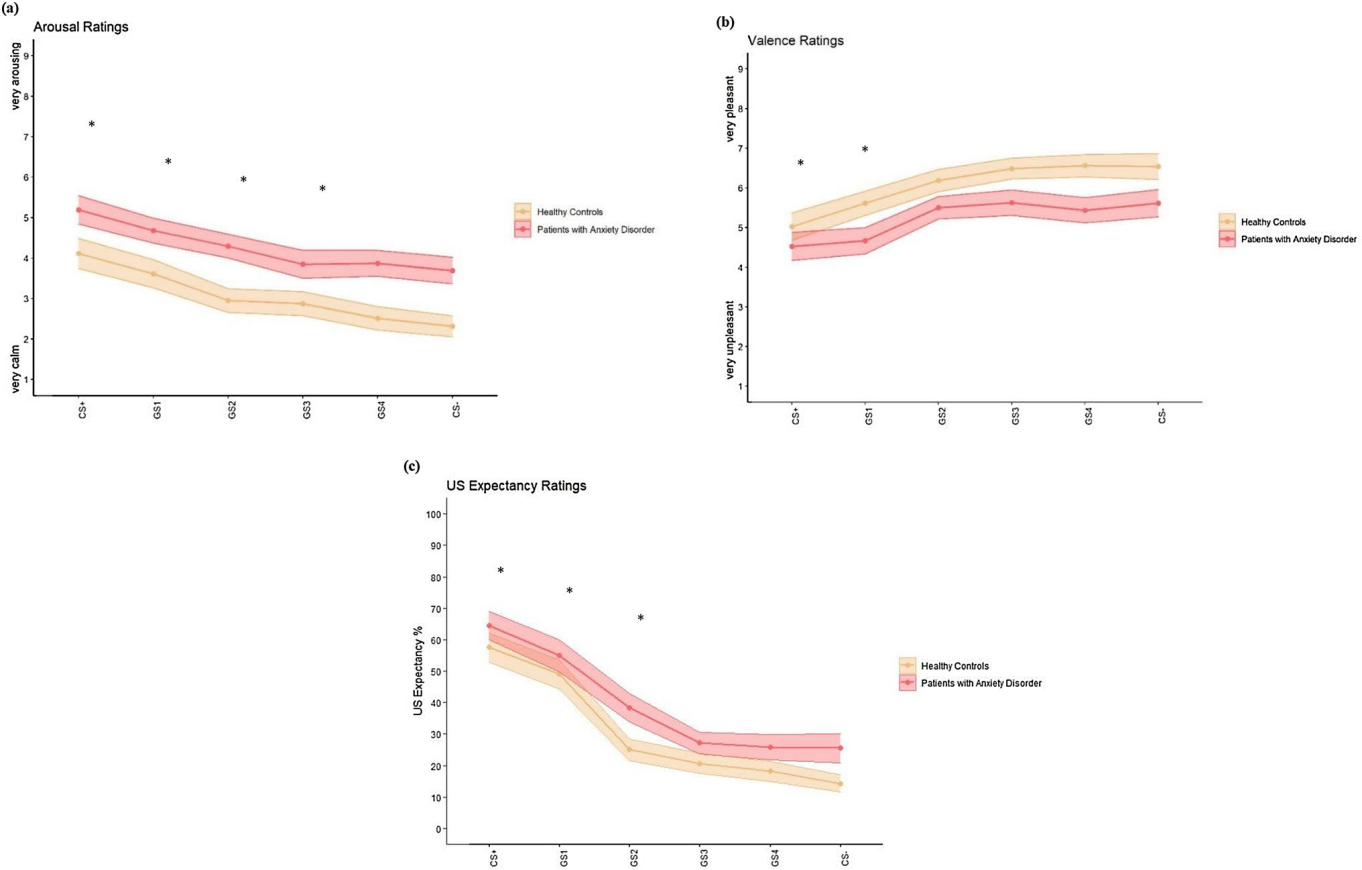
Results (means and standard error bands) during generalization for the ratings of **a** arousal, **b** valence, and **c** US expectancy to each stimulus type by group. Asterisks indicate significant differences from the reference condition CS− (*p* < 0.05)

Contrary to our assumption, there were no significant qualitative group differences in trends, as no significant stimulus type x group interaction effects were found (all *ps* ≥ 0.338). However, there were quantitative differences according to the ratings as significant main effects of group were found for all ratings (arousal: *F* (1,77) = 9.31, *p* = 0.003, *η*^2^ = 0.11; valence: *F* (1,77) = 5.02, *p* = 0.028, *η*^2^ = 0.06; US expectancy:* F* (1,77) = 4.21, *p* = 0.044, *η*^2^ = 0.05), indicating generally higher arousal and US expectancy ratings, and lower valence ratings, respectively, in anxiety patients compared to HC (Fig. 2). Thus, with respect to the ratings, both groups showed generalization of conditioned fear, but in a qualitatively “similar” way, albeit generally shifted with respect to intensity.

For the valence ratings, we found a significant phase x group interaction effect (*F* (1, 77) = 7.41, *p* = 0.008, *η*^2^ = 0.09). Post hoc tests revealed that the differences between the groups were mostly due to Generalization 1 (Generalization 1 (*t* (77) = 3.21, *p* = 0.002, Generalization 2 (*t* (77) = 1.15, *p* = 0.254) with lower valence ratings in patients (Fig. A.3).

## Discussion

The present study investigated fear acquisition and generalization in children and adolescents with clinically relevant anxiety and matched healthy controls (HC). The goal was to examine discrimination conditioning and (over)generalization in healthy vs. anxious children and adolescents to elucidate differences in fear learning and generalization between children and adolescents with anxiety disorders and HC. Therefore, the ratings of valence, arousal, and US expectancy were measured, hypothesizing that patients with clinically relevant anxiety would show overall higher fear responses during the acquisition and generalization as well as overgeneralization as indicated by more linear compared to quadratic trends when compared to HC.

These hypotheses could be partly confirmed: We found quantitative, not qualitative differences between patients and HC. In other words, patients with anxiety disorders had generally higher rating scores compared to HC, but they did not show more overgeneralization of conditioned fear when compared to HC. In the following, these results will be discussed in more detail regarding previous literature. In general, findings in some aspects are in line with results of similar experiments done in adults, but also in children [e.g., 33, 34], thereby eliciting the question if overgeneralization could be considered as risk markers for anxiety disorders or vice versa.

In line with previous experiments using the “screaming lady” paradigm in children and adults with and without clinically relevant anxiety [e.g. 25, 26, 33, 35], successful fear conditioning as well as generalization could be detected in both, patients diagnosed with an anxiety disorder and HC. Further, we found overall group differences in the ratings: children and adolescents with clinically relevant anxiety showed generally higher arousal and US expectancy ratings and more negative valence ratings to all presented stimuli during generalization phases when compared to HC. Such heightened responding to all stimuli has been attributed to associative processes of elevated responding to threat cues and poor inhibitory responding to safety cues, and non-associative processes of sensitization and habituation (see [12] for a review). Moreover, these group differences were already found during the (pre-) acquisition phases indicating a general hyper-arousal of patients with pathological anxiety. Of special interest in this context is the fact that almost half of our patient group were diagnosed with a social anxiety disorder (SAD). Thus, the general hyper-arousal of the patients might have been triggered by the exposure to disorder-relevant face stimuli. Such general hyper-arousal constitutes a diagnostic criterion of SAD in the DSM-V (American Psychiatric Association, 2013), and is in line with previous studies suggesting a generally biased processing of faces in social anxiety disorders [33, 36]. Hence, our first hypothesis, which expected that patients diagnosed with an anxiety disorder would show different ratings (higher arousal and US expectancy ratings and lower valence ratings, respectively) when compared to HC, was confirmed.

Contrary to previous findings demonstrating overgeneralization to be a diagnostic marker of some anxiety disorders in adults [15–17] and in contrast to our further hypothesis, children and adolescents with pathological anxiety compared to HC demonstrated no maladaptive overgeneralization of fear as indicated by more linear compared to quadratic trends: We found no significant differences in the generalization gradients between the groups as indicated by no significant stimulus type x group interaction effects, but general group differences as indicated by main effects of group in the arousal, valence, and US expectancy ratings: Anxious individuals reported to perceive all faces as less pleasant and more arousing, and, relative to the control group, they overestimated the pairing of GSs/ CS− with the US, although those stimuli were never reinforced.

These findings are congruent with previous findings on, e.g., face perception in social anxiety disorder (SAD) done in adults, which detected those individuals with social anxiety rated angry faces to be more negative [37] and more arousing [38], and rated happy faces as less pleasant [39]; further, socially anxious individuals showed generally enhanced US expectancy ratings when compared to controls [see 40 for a review]. This is consistent with prior studies indicating that patients with social phobia have bad stimulus discrimination skills and therefore show enhanced US expectancy ratings and fear responses to safety cues [33, 41, 42].

In line with our results, there are some further studies, which did not find clear overgeneralization in anxiety disorders [18, 19], but generally higher fear ratings in patients compared to HC [33]. Additionally, the present study investigated children and adolescents, whereas previous studies, which demonstrated overgeneralization in patients with anxiety disorders, investigated adult patients [15–17]. Relevant here is that the median onset age of anxiety disorders is about 13 years [43], with chronic manifestations and comorbidities in adulthood. Thus, healthy adults and adolescents might be those, who had not yet developed a manifest anxiety disorder during this critical time window, but which of course could possibly change later in life; thus, the adolescents in our sample may have been “healthier” than the participating children, who may still be at larger risk to develop pathological fear possibly soon and clearly before their now already adolescent counterparts. The latter would have led to their exclusion from the healthy control group (and to their inclusion into the experimental group). This means in other words that the adolescents in the control group managed to stay healthy for a longer time from their birth on than the healthy children (also due to age/time of birth), who may be prone to develop an anxiety disorder eventually quite soon considering the mean onset of about 13 years for anxiety disorders, which states a clear risk factor. As a study by Reinhard et al. [44] demonstrated, the subjective and physiological fear responses in children and adolescents aged 8 to 17 years were generally lower with increasing age irrespective of the stimulus quality. Additionally, stimulus discrimination improved with increasing age paralleled by reduced overgeneralization in older individuals. Thus, there were generally higher fear ratings in patients with AD compared to HC as well as in healthy younger children compared to healthy adolescents. Therefore, it might be important to check whether there were differences between patients with AD and healthy controls when controlling for age. Moreover, patients in the present study were very heterogeneous: Patients with any anxiety classified as pathological anxiety as well as with and without medication were included in the study. Possibly, overgeneralization is a feature of a subset of specific anxiety disorders. The experiments by Lissek et al., for instance, found overgeneralization e.g., in patients with panic disorder (PD) [15], generalized anxiety disorder (GAD) [17] and post-traumatic stress disorder (PTSD) [16]. On the other hand, a study by Ahrens et al. [33], which investigated patients with a social anxiety disorder (SAD), found that with respect to explicit ratings SAD patients compared to HC do not seem to be characterized by strong overgeneralization but discrepancies in fear responses to both conditioned and generalized threat stimuli. As mentioned before, most patients in our sample were diagnosed with SAD. Further, due to the fact, that we investigated children and adolescents rather than adults, there were hardly any participants with PD/GAD/PTSD in our sample. Thus, this fits to previous studies showing that due to ratings there were parallel shifted gradients rather than qualitative differences in fear generalization between SAD patients and HC [33]. Additionally, in the experiments of Lissek and colleagues [15–17], shocks were used as aversive stimulus, whereas in our experiment, a loud scream was used, and further, different dependent variables (e.g., startle response vs. SCR/ratings), which possibly reduces the direct comparability of the results.

Despite its strengths, the current study was not without limitations. First, the sample size was relatively small and we were unable to investigate possible differences between patients with different types of anxiety disorders. Moreover, due to the relatively small sample, we did our analyses not separated by sex, but differences relating to sex could be quite interesting, considering that the prevalence of anxiety disorders is supposed to be significantly higher for females than for males [45, 46, ICD 10: WHO 2004]. Further, participants received different medication, which we have not checked for as a covariate. Additionally, we did not control for comorbid disorders, and thus, we cannot rule out that results were distorted by other psychiatric disorders. A study in adults, for instance, investigated the influence of comorbid depression on social phobia and demonstrated that patients without depression showed defensive hyper-reactivity during social threat imagery, while patients with comorbid depression showed attenuated reactions [47]. This could reflect depression-associated psychomotor retardation and behavioral inhibition. On the other side, most of the other studies on fear generalization included also individuals with comorbid disorders, but mostly with other anxiety disorders or depression [15, 17]. Second, we analyzed group differences in a categorical, not dimensional manner leaving the possibility out of consideration, that even supposedly healthy participants and/or subclinical groups at risk for developing anxiety disorders could have high anxiety scores when measured by, e.g., the State-Trait Anxiety Inventory for Children – Trait version (STAIC-T [30]) [see e.g., 48–50]. Third, other factors accounting for anxiety disorders as well as resilience factors were disregarded here. For instance, additional risk factors, such as child temperament (e.g., neuroticism), parental anxiety disorder, parenting style, and/or negative life events [51, 52] might play an important role but were not assessed in this study. Moreover, of note is the fact, that our study has a cross-sectional design. Thus, the question remains still open, if quantitative/qualitative differences during the aversive conditioning and generalization found in patients with an anxiety disorder are really a risk marker or rather a result of the disorder. Thus, longitudinal follow-up studies are required to answer this still-open question. This question is highly important, especially with respect to preventive and therapeutic approaches. Since there are therapeutic interventions accessible to date, but several children do not improve, new markers could lead the way to a “personalized medicine approach “.

In sum, the results of the present study indicate that patients with clinically relevant anxiety differed from healthy control children in terms of responding during aversive conditioning and generalization. The differences, however, were more quantitative, not qualitative. In other words, the patients showed generally higher fear-relevant ratings when compared to HC, suggesting elevated responding to the threat cue and impaired inhibition of responses to the safety cues. Thus, according to our results, overgeneralization of fear does not seem to be a diagnostic marker of anxiety disorders in children and adolescents. There is a clear need for replication, especially in bigger and better-classified samples. Moreover, there is a clear need for longitudinal studies to answer the question if (over)generalization patterns of patients with anxiety disorders are a risk factor for any anxiety disorder or if certain patient groups develop such generalization pattern as part of the disorder. As the present study demonstrated that the fear generalization pattern of children is not necessarily comparable with the generalization pattern of adults, developmental factors need to be considered when analyzing fear learning and generalization in anxiety disorders.

### Supplementary Information

Below is the link to the electronic supplementary material.

Supplementary file1 (DOCX 1058 KB)

## Data Availability

The data that support the findings of this study are available from the corresponding author upon request.
