# Supplementary material for: Fear conditioning and fear generalization in children and adolescents with anxiety disorders
Source: Eur Child Adolesc Psychiatry. 2023 Oct 4;33(7):2163–72. doi: 10.1007/s00787-023-02304-7 (PMC11255079; doi:10.1007/s00787-023-02304-7)
Supplement: Supplementary file 1 — Supplementary file1 (DOCX 1058 KB) [file 787_2023_2304_MOESM1_ESM.docx]

Supplementary Material

European Child & Adolescent Psychiatry

Fear conditioning and fear generalization in children and adolescents with anxiety disorders

Julia Reinhard^1*^, Anna Mittermeier^1^, Lisa Brandstetter^1^, Kimberly Mowat^1^, Anna Slyschak^1^, Andrea M.F. Reiter^1^, Matthias Gamer^2^, Marcel Romanos^1^

^1^ Center of Mental Health, Department of Child and Adolescent Psychiatry, Psychosomatics and Psychotherapy, University Hospital of Würzburg, Germany

^2^ Department of Psychology (Experimental Clinical Psychology), University of Würzburg, Würzburg, Germany.

*Corresponding author: Email: [Reinhard_J@ukw.de](mailto:Reinhard_J@ukw.de)

|  | *Stimulus Type* | *Phase* | *Stimulus Type x Phase* |
| --- | --- | --- | --- |
| **Arousal** | *F* (1,77) = 25.75,  *p* < .001, *η*^2^ = .25 | *F* (2,142) = 11.33,  *p* < .001, *η*^2^ = .13 | *F* (2,154) = 12.28,  *p* < .001, *η*^2^ = .14 |
| **Valence** | *F* (1,77) = 16.00,  *p* < .001, *η*^2^ = .17 | *F* (2,127) = 2.61,  *p* = .088, *η*^2^ = .03 | *F* (2,137) = 12.09,  *p* < .001, *η*^2^ = .14 |
| **US Expectancy** | *F* (1,77) = 51.15,  *p* < .001, *η*^2^ = .40 | *F* (2,123) = 21.60,  *p <* .001, *η*^2^ = .22 | *F* (2,154) = 34.00,  *p* < .001, *η*^2^ = .31 |

**Table A.1. Results of ANOVAs at pre- acquisition/acquisition.** Main effects of stimulus type and phase as well as their interaction effects on arousal, valence, and US expectancy ratings

**Table A.2. Descriptive Statistics at pre- acquisition (PA)/acquisition (A)** on arousal, valence, and US expectancy ratings

| **Arousal** | **PA_CS-** | **PA_CS+** | **A_CS-_1** | **A_CS+_1** | **A_CS-_2** | **A_CS+_2** | | |
| --- | --- | --- | --- | --- | --- | --- | --- | --- |
| Mean | 3.29 | 3.20 | 3.49 | 4.44 | 3.43 | 4.66 | | |
| Std. Deviation | 1.84 | 1.85 | 2.08 | 2.30 | 2.13 | 2.33 | | |
| **Valence** | **PA_CS-** | **PA_CS+** | **A_CS-_1** | **A_CS+_1** | **A_CS-_2** | **A_CS+_2** |  |  |
| Mean | 5.66 | 5.80 | 5.79 | 5.18 | 5.92 | 4.79 |  |  |
| Std. Deviation | 1.92 | 1.87 | 1.89 | 2.05 | 2.14 | 2.19 |  |  |
|  |  |  |  |  |  |  |  |  |
| **US Expectancy** | **PA_CS-** | **PA_CS+** | **A_CS-_1** | **A_CS+_1** | **A_CS-_2** | **A_CS+_2** | |  |
| Mean | 4.63 | 4.39 | 4.90 | 6.96 | 4.01 | 7.94 | |  |
| Std. Deviation | 2.78 | 2.82 | 2.75 | 2.86 | 3.10 | 3.04 | |  |

**Table A.3. Results of ANOVAs at generalization.** Main effects of stimulus type and trends of arousal, valence, and US expectancy ratings

|  | *Stimulus Type* | *linear* | *quadratic* |
| --- | --- | --- | --- |
| **Arousal** | *F* (3,231) = 27.64,  *p* < .001, *η*^2^ = .26 | *F* (1,77) = 54.93,  *p* < .001, *η*^2^ = .42 | *F* (1,77) = 5.86,  *p =* .018, *η*^2^ = .07 |
| **Valence** | *F* (3,240) = 18.33,  *p* < .001, *η*^2^ = .19 | *F* (1,77) = 34.69  *p <* .001, *η*^2^ = .31 | *F* (1,77) = 9.60,  *p =* .003, *η*^2^ = .11 |
| **US Expectancy** | *F* (2,187) =65.38,  *p* < .001, *η*^2^ = .46 | *F* (1,77) = 106.12  *p <*.001, *η*^2^ = .58 | *F* (1,77) = 32.23  *p <* .001, *η*^2^ = .30 |

**Table A.4. Descriptive Statistics at generalization (GEN)** on arousal, valence, and US expectancy ratings

| **Arousal** | **GEN_CS+** | | | **GEN_CS-** | | | **GEN_GS1** | | | **GEN_GS2** | | | **GEN_GS3** | | | **GEN_GS4** | | |  |  |
| --- | --- | --- | --- | --- | --- | --- | --- | --- | --- | --- | --- | --- | --- | --- | --- | --- | --- | --- | --- | --- |
| Mean | 4.65 | | | 2.99 | | | 4.14 | | | 3.61 | | | 3.35 | | | 3.18 | | |  |  |
| Std. Deviation | 2.33 | | | 1.97 | | | 2.12 | | | 1.96 | | | 2.05 | | | 2.02 | | |  |  |
| **Valence** | | **GEN_CS+** | | | **GEN_CS-** | | | **GEN_GS1** | | | **GEN_GS2** | | | **GEN_GS3** | | | **GEN_GS4** | | |  |
| Mean | | 4.78 | | | 6.08 | | | 5.15 | | | 5.85 | | | 6.06 | | | 6.01 | | |  |
| Std. Deviation | | 2.19 | | | 2.15 | | | 2.05 | | | 1.80 | | | 1.87 | | | 1.95 | | |  |
| **US Expectancy** | | | **GEN_CS+** | | | **GEN_CS-** | | | **GEN_GS1** | | | **GEN_GS2** | | | **GEN_GS3** | | | **GEN_GS4** | | |
| Mean | | | 7.10 | | | 2.98 | | | 6.19 | | | 4.16 | | | 3.39 | | | 3.19 | | |
| Std. Deviation | | | 2.89 | | | 2.42 | | | 3.03 | | | 2.57 | | | 2.12 | | | 2.28 | | |

**Fig. A.1**

**Fig1.** Schematic overview of the fear conditioning and generalization paradigm. During Pre-Acquisition phase four CS+/CS- were presented. During Acquisition phases 12 CS+/CS- were presented. During Generalization phases 12 CS+/CS- as well as 12 GS1/GS2/GS3/GS4, resulting in 72 stimuli during generalization phases.

**Fig A.2**

**(a)**


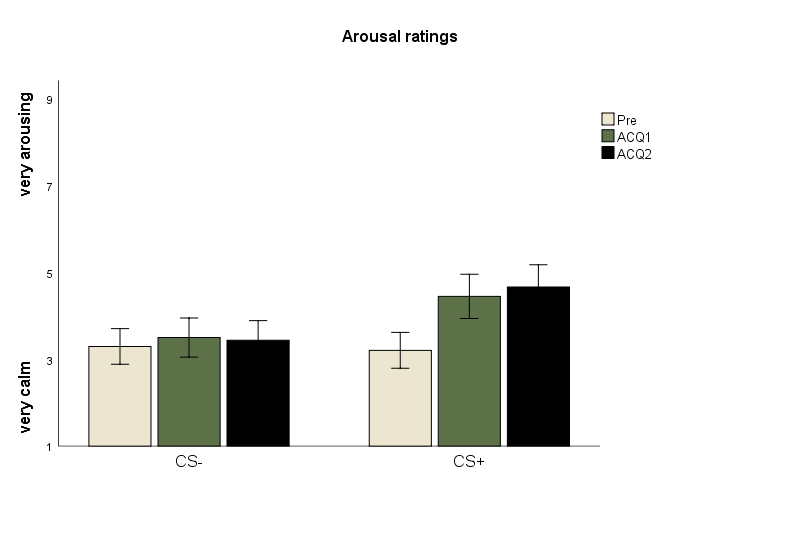


*

*

**(b)**


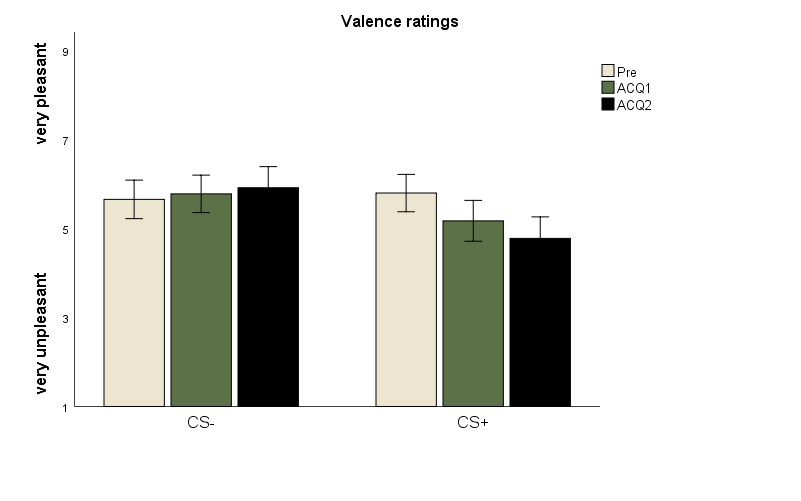


*

*

**(c)**


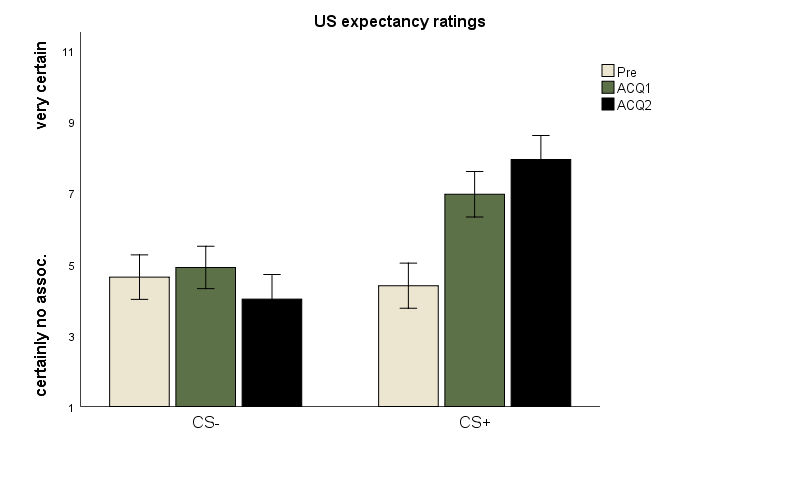


*

*

*

*

**Fig2.** Average ratings (with standard errors) of (a) arousal, (b) valence, and (c) US expectancy to CS- and CS+ after pre-acquisition (Pre), Acquisition 1 (ACQ 1) and Acquisition 2 (ACQ 2). Asterisks indicate significant differences between stimuli (p<.001).

**Fig A.3**


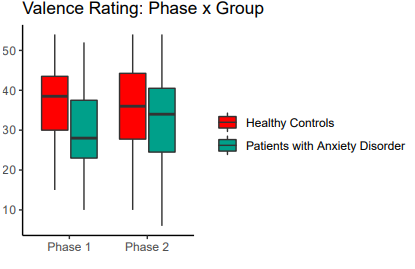


*

Valence Ratings

**Fig3.** Phase x group interaction effect for valence ratings. The lower and upper hinges correspond to the first and third quartiles (i.e., the 25th and 75th percentiles). The horizontal line shows the median value. Whiskers (vertical lines) extend from the hinge to the lowest/largest individual data point, no further than 1.5 * inter-quartile ranges from the hinge. Asterisks indicate significant group differences (p<.01).
